# KLF4-Induced Connexin40 Expression Contributes to Arterial Endothelial Quiescence

**DOI:** 10.3389/fphys.2019.00080

**Published:** 2019-02-12

**Authors:** Jean-François Denis, Mannekomba R. Diagbouga, Filippo Molica, Aurélie Hautefort, Tanja Linnerz, Masakatsu Watanabe, Sylvain Lemeille, Julien Y. Bertrand, Brenda R. Kwak

**Affiliations:** ^1^Department of Pathology and Immunology, University of Geneva, Geneva, Switzerland; ^2^Graduate School of Frontier Biosciences, Osaka University, Osaka, Japan; ^3^Department of Medical Specializations – Cardiology, University of Geneva, Geneva, Switzerland

**Keywords:** endothelium, shear stress, KLF4, Cx40, cell proliferation

## Abstract

Shear stress, a blood flow-induced frictional force, is essential in the control of endothelial cell (EC) homeostasis. High laminar shear stress (HLSS), as observed in straight parts of arteries, assures a quiescent non-activated endothelium through the induction of Krüppel-like transcription factors (KLFs). Connexin40 (Cx40)-mediated gap junctional communication is known to contribute to a healthy endothelium by propagating anti-inflammatory signals between ECs, however, the molecular basis of the transcriptional regulation of Cx40 as well as its downstream effectors remain poorly understood. Here, we show that flow-induced KLF4 regulated Cx40 expression in a mouse EC line. Chromatin immunoprecipitation in ECs revealed that KLF4 bound to three predicted KLF consensus binding sites in the Cx40 promoter. HLSS-dependent induction of Cx40 expression was confirmed in primary human ECs. The downstream effects of Cx40 modulation in ECs exposed to HLSS were elucidated by an unbiased transcriptomics approach. Cell cycle progression was identified as an important downstream target of Cx40 under HLSS. In agreement, an increase in the proportion of proliferating cell nuclear antigen (PCNA)-positive ECs and a decrease in the proportion of ECs in the G0/G1 phase were observed under HLSS after Cx40 silencing. Transfection of communication-incompetent HeLa cells with Cx40 demonstrated that the regulation of proliferation by Cx40 was not limited to ECs. Using a zebrafish model, we finally showed faster intersegmental vessel growth and branching into the dorsal longitudinal anastomotic vessel in embryos knock-out for the Cx40 orthologs *Cx41.8* and *Cx45.6*. Most significant effects were observed in embryos with a mutant *Cx41.8* encoding for a channel with reduced gap junctional function. Faster intersegmental vessel growth in *Cx41.8* mutant embryos was associated with increased EC proliferation as assessed by PH3 immunostaining. Our data shows a novel evolutionary-conserved role of flow-driven KLF4-dependent Cx40 expression in endothelial quiescence that may be relevant for the control of atherosclerosis and diseases involving sprouting angiogenesis.

## Introduction

Arteries are exposed to various biomechanical forces, which they sense via numerous mechanoreceptors (Kwak et al., 2014). On the luminal side, endothelial cells (ECs) sense shear stress, a blood flow-induced frictional force. This force varies in time, magnitude and direction according to the pulsatility of blood flow and vessel geometry, and is a critical regulator of endothelial function (Davies et al., 2013; Simmons et al., 2016). Atherosclerosis predominantly develops in regions of the arterial tree that are subjected to disturbed blood flow such as arterial bifurcations (Brown et al., 2016; Morbiducci et al., 2016). In contrast, straight parts of arteries experiencing a high laminar shear stress (HLSS) are protected from the disease. ECs in athero-susceptible regions are prone to pro-inflammatory activation and display an elevated permeability to lipoproteins compared to ECs at athero-protected sites (Kwak et al., 2014). Possibly as a response to injury, ECs at regions of disturbed flow regions display an increased turnover (Kwak et al., 2014).

Members of the Krüppel-like family of transcription factors, i.e., KLF2 and KLF4, are highly expressed in regions exposed to athero-protective flow (Tabas et al., 2015). They take part in the anti-inflammatory phenotype of ECs through induction of endothelial nitric oxide synthase expression and by attenuating the expression of the vascular cell adhesion molecule-1 and E-selectin, for example (SenBanerjee et al., 2004; Dekker et al., 2005). Although ECs display substantial heterogeneity in their individual response to receptor-mediated signaling (Huang et al., 2000; Pogoda et al., 2018), the presence of gap junctions between high and low responders enables a synchronized response with the endothelial syncytium.

Gap junction channels are comprised of connexins (Cxs), a family of transmembrane proteins with high homology among vertebrates, in particular in the N-terminal-, transmembrane- and extracellular loop domains (Eastman et al., 2006; Cruciani and Mikalsen, 2007). There are 20–21 Cxs in mammals and the zebrafish genome has been predicted to contain 39 Cx-encoding genes (Watanabe, 2017). This high number of Cxs can be attributed to gene duplication during fish evolution, but it is not known whether all the genes are expressed and functional (Watanabe, 2017). Six Cxs oligomerize to form a connexon, and two connexons from adjacent cells dock to establish a gap junction channel, enabling direct communication between neighboring cells through diffusion of ions and small metabolites (Leybaert et al., 2017). ECs in arteries express Cx37, Cx40, and Cx43 in a non-uniform manner (Molica et al., 2018). Cx43 is predominantly expressed in ECs at branching points or bifurcations of arteries exposed to disturbed blood flow (Gabriels and Paul, 1998), whereas Cx37 and Cx40 are expressed in ECs of straight arterial parts exposed to HLSS (Pfenniger et al., 2012; Denis et al., 2017). Interestingly, endothelial Cx40 interacts with IκBα, resulting in decreased activation of the NF-κB transcription factor thereby limiting the inflammatory response (Denis et al., 2017). In agreement, EC-specific deletion of Cx40 accelerates atherosclerosis in mice (Chadjichristos et al., 2010).

Little is known regarding the signal transduction pathways involved in HLSS-dependent regulation of Cx40 levels. A low level of laminar shear stress (LLSS) has been shown to induce a long-term expression of Cx40 in human umbilical vein ECs with PI3K activity being required for basal Cx40 expression and Akt activity taking part in its shear stress-dependent induction (Vorderwulbecke et al., 2012). However, the transcriptional regulation of Cx40 induction by arterial HLSS levels remains to be investigated. Moreover, no large-scale analysis to elucidate the downstream signaling and function of Cx40 under arterial shear stress levels has been performed to date.

## Materials and Methods

### Cell Culture

Different cell lines were used: (a) a mouse EC line (bEnd.3) (Kwak et al., 2001), (b) a communication-incompetent sub-clone of human HeLa cells (ATCC), and (c) the afore-mentioned HeLa cells stably transfected with murine Cx40 (Elfgang et al., 1995). Cells were grown in DMEM (41966-029, Gibco) supplemented with 10% fetal bovine serum (FBS; Sigma), 5000 U/L penicillin and 5 mg/mL streptomycin (Gibco). The medium of the stably Cx40-transfected HeLa cells was supplemented with 0.5 μg/mL puromycin (Sigma-Aldrich). bEnd.3 cells were grown on coverslips, dishes or Ibidi μ-Slide VI^0.4^ coated with 1.5% gelatin (Sigma-Aldrich).

Silencing of KLF4 or Cx40 in bEnd.3 cells was done using short interfering RNA (siRNA) SMARTpool ON-TARGETplus Mouse KLF4 siRNA or Mouse Gja5 siRNA (Dharmacon). The ON-TARGETplus Non-targeting (NT) Pool (Dharmacon) was used as negative control. The transfections were performed under serum-free conditions with 25 nmol/L siRNA and 5 mg/L DharmaFECT4 (Dharmacon). The efficiency of the KLF4 silencing was determined by quantitative real-time PCR (qPCR).

Human umbilical vein endothelial cells (HUVECs) from two different donors were purchased from Lonza and grown in EGM-2 medium (Promocell) at 37°C in a humidified atmosphere containing 5% CO_2_. Cells were passaged by trypsin–EDTA (Gibco) treatment and used at passages 4–6 for each donor.

### Flow Experiments

Sixty thousand bEnd.3 cells were seeded per Ibidi μ-Slide VI^0.4^ channel and grown till confluence. Thereafter, siRNA for Cx40 or KLF4 was applied for 24 h as detailed above. A non-targeting pool of siRNA (siNT) was used as a control. Thereafter, cells were exposed for 48 h to defined flow conditions, i.e., HLSS: 20 dynes/cm^2^, LLSS: 5 dynes/cm^2^ or oscillatory shear stress (OSS): 5 dynes/cm^2^, 1 Hz, using the Ibidi pump system. Control cells were kept under no flow (static) conditions.

Alternatively, bEnd.3 cells or HUVECs were seeded in 6-well plates and grown to confluence. Thereafter, the 6-well plates were placed onto an orbital rotating platform (Grant Instruments) housed inside a cell culture incubator, and ECs were cultured for an additional 48 h. The radius of orbit of the orbital shaker was 10 mm and the rotation rate was set to 210 rpm, which induced swirling of the 3 mL culture medium over the cell surface and exposed the ECs to a wall shear stress of a defined magnitude and direction at the base, as described previously (Warboys et al., 2014). ECs exposed to undisturbed flow (HLSS; at the periphery) and disturbed flow (OSS; in the center) were compared to static conditions.

### Immunofluorescence Staining

Immunofluorescent staining on bEnd.3 cells was performed as previously described (Kwak et al., 2001). In short, confluent bEnd.3 cultures were fixed with 100% methanol at -20°C for 5 min. Subsequently, the samples were permeabilized with 0.2% Triton X-100 in PBS for 30 min, charges neutralized with 0.5 mol/L NH_4_Cl in PBS for 15 min and blocked with 2% bovine serum albumin (BSA; AppliChem) or with 10% normal goat serum (Vector Laboratories) in PBS for 30 min. Next, the primary antibody against Cx40 (Alpha-Diagnostics; 1/200) or anti-proliferating cell nuclear antigen (PCNA; Thermo Fisher Scientific; 1/25) in blocking solution was applied and incubated overnight at 4°C. Finally, an Alexa Fluor 488-conjugated goat anti-rabbit antibody (Thermo Fisher Scientific, 1/2000) was used for signal detection. The nuclei and cells were counterstained with 4^′^,6-diamidino-2-fenylindool (DAPI) and 0.003% Evans Blue, respectively. Samples on coverslips were mounted with Vectashield (Vector Laboratories) or with Ibidi mounting medium for Ibidi μ-Slides and imaged with an epifluorescent Zeiss Axiocam fluo microscope (Zeiss Axio Imager Z1) equipped with an AxioCam 506 mono camera (Carl Zeiss AG) and analyzed using the Zeiss Zen 2.3 software.

### Fluorescence-Activated Cell Sorting (FACS)

Cell cycle status was determined in bEnd.3 cells using Hoechst 33342 (Thermo Fisher Scientific), a non-toxic specific vital stain for DNA, at a concentration of 10 μg/mL in Ibidi slides. Thereafter, cells were detached with trypsin (Gibco) for 5 min and their distribution was measured using a BDLSR Fortessa FACS (Becton Dickinson) equipped with a BD HTS unit (Becton Dickinson). Analysis of the cell populations in the different cell cycle compartments was performed using FlowJo software.

### Western Blot

Cell cultures were rinsed with PBS, pH = 7.4, and lysed in RIPA buffer as previously described (Pfenniger et al., 2012). After protein concentration quantification with a Micro BCA protein assay kit (Thermo Scientific), 5 μg (HUVECs) or 10 μg (bEnd.3) of protein was separated by SDS-PAGE and transferred to PVDF-membrane (Immobilon, Millipore). After 2 h blocking with 5% milk and 1% Tween in PBS, the membrane was exposed to anti-Cx40 (Alpha-Diagnostics, 1/500) or anti-β-actin (Sigma, 1/10000) primary antibodies in blocking solution. Revelation was performed by incubating the membrane for 1 h at RT with secondary horseradish peroxidase-conjugated antibodies (Jackson ImmunoResearch; 1/5000) and followed by ECL detection (Millipore) using ImageQuant LAS 4000 software. Band intensities were thereafter quantified using ImageJ software. Cx40 results were normalized to β-actin.

### Quantitative Real-Time PCR

Total RNA was extracted from bEnd.3 cells using the NucleoSpin RNA II kit (Macherey-Nagel) according to the manufacturers’ instructions. After extraction, RNA concentration was determined using a Nanodrop 2000c (Thermo Fisher Scientific) and equal amounts of RNA were used to obtain cDNA with the reverse transcriptase reaction kit (QuantiTect Reverse Transcription Kit, Qiagen). PCR was performed using the ABI StepOne Plus detection system with Taqman gene expression assays (Applied Biosystems) according to the manufacturers’ protocol. All reactions were normalized to GAPDH.

### Chromatin Immunoprecipitation (ChIP)

bEnd.3 cells were grown until confluence in P100 dishes. The Simple Chip Enzymatic Chromatin IP kit with magnetic beads (Cell Signaling Technology) was used according to the manufacturers’ protocol. In short, chromatin was cross-linked with formaldehyde (Thermo Fisher Scientific) to a final concentration of 1% for 10 min and the reaction was stopped by adding glycine. Next, the cells were lysed and nuclei extracted. Micrococcal nuclease and sonication were used to fragment DNA (fragmentation between 150 and 900 bp) and cross-linked chromatin was extracted. The efficiency of the chromatin digestion was determined by electrophoresis on a 1% agarose gel. Ten micrograms of digested, cross-linked chromatin was immunoprecipitated using 2 μg anti-KLF4 antibody (R&D systems) and protein magnetic beads. As positive control a monoclonal rabbit anti-Histone H3 antibody (Cell Signaling Technology) was used and as negative control a rabbit IgG (Cell Signaling Technology). Finally, after elution and the reversal of the cross-linking, DNA was purified and efficiency of the procedure was assessed by qPCR. Putative KLF consensus binding sites in the promoter region of Cx40 [CACCC elements and other probable transcription factor binding sites (TFBS)] were predicted using the MatInspector online software^1^. Primers for the Cx40 promoter region were designed using the Primer3 version 0.4.0 online software. Primer sequences, named according to the location of the TFBS with respect to the transcription start site, are as follows:

- 2477 forward: AGCTTCTTTGCAGTGCCATT,
- 2477 reverse: TGCCATGCTCTCTCCTTCTT;
- 2599 forward: CCATCTCACCAGCCCTAAAG,
- 2599 reverse: TGGGCACACTTCACAATGTT;
- 4196 forward: TCTCAACCAGCAGAAACGTG,
- 4196 reverse: ATGGCAACACGTGCAAGTAA.

As KLF4 positive control, primers against a previously known TFBS on the fibroblast-specific protein 1 (FSP1) promoter region were used (Cuttano et al., 2016). Quantification of the ChIP efficiency was performed by qPCR with Power Up SYBR Green Master Mix (Applied Biosystems).

### Library Preparation, Sequencing, Read Mapping to the Reference Genome and Gene Coverage Reporting

Following the above-described flow conditions, total RNA was isolated for the four conditions using the NucleoSpin RNA II kit (Macherey-Nagel) according to the manufacturers’ instructions. Quality of the three samples per condition was verified using the Agilent 2100 Bioanalyzer with the Agilent RNA 6000 Nano Kit (Agilent Technologies). cDNA libraries were constructed by the Genomic platform of the University of Geneva using the Illumina TruSeq RNA sample Preparation Kit according to the manufacturers’ protocol. Libraries were sequenced using single-end (50 nt-long) on Illumina HiSeq2000. FastQ reads were mapped to the ENSEMBL reference genome (GRCm38.80) using STAR version 2.4.0j (Dobin et al., 2013) with standard settings, except that any reads mapping to more than one location of the genome (ambiguous reads) were discarded (*m* = 1). Sequence data have been submitted to the GEO database under accession number GSE118717. A unique gene model was used to quantify reads per gene. In short, the model considers all annotated exons of all annotated protein coding isoforms of a gene to create a unique gene where the genomic region of all exons are considered as coming from the same RNA molecule and merged together.

### RNAseq Analysis

All reads overlapping the exons of each unique gene model were reported using featureCounts version 1.4.6-p1 (Quinlan and Hall, 2010). Gene expressions were reported as raw counts and normalized in reads per kilobase per million (RPKM) in order to filter out genes with low expression value (<1 RPKM) before calling for differentially expressed genes. Library size normalizations and differential gene expression calculations were performed using the package edgeR (Robinson et al., 2010) designed for R software (Team, 2008). Only genes having significant fold-change (Benjamini-Hochberg corrected *p*-value <0.01) were considered for the rest of the RNAseq analysis. Differentially expressed genes were categorized on basis of their involvement in atherosclerosis-related processes such as inflammation, angiogenesis, permeability, vascular tone, extracellular matrix (ECM) interaction, metabolic processes or immune response. In each condition, approximately 50% of differentially expressed genes did match to these categories.

### Gene Set Enrichment Analysis (GSEA)

All annotated pathways for *Homo sapiens, Mus musculus, Rattus norvegicus* and *Danio rerio* available on WikiPathways database^2^ were used to generate gene sets, as well as the KEGG metabolic pathways (KEGG^3^) relative to GRCm38.80. Genes were ranked by calculated fold-changes (decreasing ranking). A gene set analysis using the GSEA package Version 2.2 (Mootha et al., 2003; Subramanian et al., 2005) from the Broad Institute (MIT, Cambridge, MA, United States) was used to analyze the pattern of differential gene expression between the two groups. Gene set permutations were performed 1,000 times for each analysis. The Normalized Enrichment Score (NES) was calculated for each gene set. GSEA results with a nominal fold discovery rate (FDR) < 0.05 and abs(NES) > 1 were considered significant. In order to identify the subset of genes that are involved in several biological processes, a leading-edge analysis was performed. All leading-edge subsets of each significant regulated pathway [FDR < 0.05 and abs(NES) > 1] were extracted and the number of times each gene appears was reported. Then, per pathway, the mean of gene occurrences was calculated and, in order to simplify the picture, pathways with a mean lower than 10% of the total number of regulated pathways were removed from the analysis. Next, the counts of each gene were re-calculated and leading-edge genes as well as remaining pathways were hierarchically clustered on an heatmap. Only genes with a total number of occurrences higher than 2 appear on the heatmap. Per comparison, pathways involving up- or down-regulated genes were analyzed separately.

### Zebrafish Strains, Husbandry, and Genotyping

AB^∗^ zebrafish strains, along with transgenic and leopard mutant strains, were kept in a 14/10 h light/dark cycle at 28°C. Embryos were obtained as described previously (Westerfield, 2000). We used the following strains: *Tg(flk1:eGFP)^s843^* (Jin et al., 2005), *sih^tc300b^* (Chen et al., 1996), *leo^t1/t1^* (hereafter called: *Cx41.8^t1/t1^*) (Watanabe et al., 2006), *leo^tq270/tq270^* (hereafter called: *Cx41.8^tq/tq^*) (Watanabe et al., 2006), *Cx41.8^t1/t1^Cx45.6^-/-^, Tg(flk1:eGFP)Cx41.8^t1/t1^, Tg(flk1:eGFP)Cx41.8^tq/tq^*, and *Tg(flk1:eGFP)Cx41.8^t1/t1^Cx45.6^-/-^.* The *Cx45.6* knock-out mutant was generated by Transcription Activator-Like Effector Nucleases (TALENs) technique using a similar procedure as previously described (Misu et al., 2016). The target sequence for the *Cx45.6* knock-out was 5^′^-TGTCTGTTATGACCGAGCTTTccccattgctcatatccGCTACTGGGTGCTGCAGA-3^′^ (TALEN binding sequences are shown in upper case and spacer sequence in lower case).

For genotyping genomic DNA was extracted from fin clips and used for PCR using the following primer pairs:

forward Cx41.8^t1^: AGATCAGAGAAGGTGTAGAC,
reverse Cx41.8^t1^: AGGTTAATTGGGCAAATTAGG;
forward Cx41.8^tq^: TGCTGCAAACATACGTCCTC,
reverse Cx41.8^tq^: TTTGCAGAGTTCTGCTGGTG;
forward Cx45.6: GGTGAGGAGTATGGGGGACT,
reverse Cx45.6: AGGGTGTCGATACGAAGACG.

The t1 and tq270 mutations in the *Cx41.8* gene were detected by sequencing of the PCR fragments. Of note, the C>T t1 mutation results in premature stop codon and a knock-out phenotype. The A>T tq270 mutation results in a reduced channel function (Watanabe et al., 2006, 2016). Cx45.6 PCR fragments were digested with BsrDI resulting in 221 bp and 54 bp fragments for the wild-type allele and a 268 bp fragment for the mutant allele.

### Cell Sorting and qPCR

Zebrafish embryos [24–48 h-post-fertilization (hpf)] were incubated in 0.5 mg/mL Liberase (Roche) solution for 90 min at 33°C, then dissociated and resuspended in 0.9X PBS-1% FBS. We excluded dead cells by SYTOX-red (Life Technologies) staining. Cell sorting for eGFP was performed using an Aria II (BD Biosciences).

RNA from eGFP^+^ and eGFP^-^ populations was extracted using the NucleoSpin RNA II kit (Macherey-Nagel) and reverse transcribed into cDNA using the QuantiTect Reverse Transcription Kit (Qiagen). qPCR was performed using the ABI StepOne Plus detection system with SYBR green gene expression assays (KAPA SYBR FAST, Kapa Biosystems) supplemented with ROX high detection solution according to the manufacturers’ protocol using the following primers:

forward Cx41.8: ACCGAGGTTGAATGCTCC,
reverse Cx41.8: TGGTTTCAATCAGGCTCC;
forward Cx45.6: CTAAGCCTGCGCTTGTCTCT,
reverse Cx45.6: GGCTCGGGTTCGAAGTGAAA;
forward Ef1α: GGTAGTATTTGCTGGTCTCG,
reverse Ef1α: GAGAAGTTCGAGAAGGAAGC.

Each qPCR experiment was performed using biological triplicates. Experiments were repeated three times and fold-change averages from each experiment were obtained.

### Zebrafish Embryo Imaging

Transgenic fluorescent embryos were dechorionated with pronase (Sigma-Aldrich) at 24 hpf and held in E3 buffer containing 0.003% tricaine methanesulfonate (Sigma-Aldrich) for imaging. Imaging was performed at 24, 28 or 48 hpf using an Olympus IX83 microscope and processed using CellSens Dimensions software.

The developmental stage of intersegmental vessels (ISVs) was quantified on the 10 proximal ISVs of the dorsal aorta (DA) in each individual fish at 28 hpf. A blinded observer classified the ISVs as: (1) partial ISV sprouts, (2) complete (full-length) ISVs, or (3) ISVs with complete branching forming the dorsal longitudinal anastomotic vessel (DLAV). Scoring distribution was compared with Fisher’s exact test.

Zebrafish embryos at 24 hpf were overnight stained with rabbit anti-PH3 (Abcam; 1/250) and chicken anti-eGFP (Life Technologies; 1/1400) after a 4-h fixation with 4% PFA at 4°C. Embryos were thereafter washed with 0.02% Triton X-100 in PBS and blocked for 1 h at 4°C in the same solution containing 4% BSA and 0.02% NaN_3_. Embryos were then incubated overnight at 4°C. After washing with 0.01% Triton X-100 in PBS, embryos were incubated with appropriate secondary antibodies [Alexa Fluor 488-conjugated anti-chicken (Life Technologies, 1/1000) and AlexaFluor594-conjugated anti-rabbit (Life Technologies, 1/1000)] and washed again before imaging with an Olympus IX83 microscope and processed using CellSens Dimensions software. A blinded observer counted the PH3^+^ nuclei in the proximal 10 ISVs of each embryo.

### Statistics

Unless otherwise specified, results are presented as mean ± SEM. Unpaired Student’s *t*-tests (equal variance), Mann–Whitney *U* tests (unequal variance) or one-way ANOVAs were used to compare differences between groups. Differences with a *P* < 0.05 were considered statistically significant; ^∗^*P* < 0.05; ^∗∗^*P* < 0.01; ^∗∗∗^*P* < 0.001; ^∗∗∗∗^*P* < 0.0001.

## Results

### KLF4 Regulates Flow-Dependent Cx40 Expression

**FIGURE 1:**
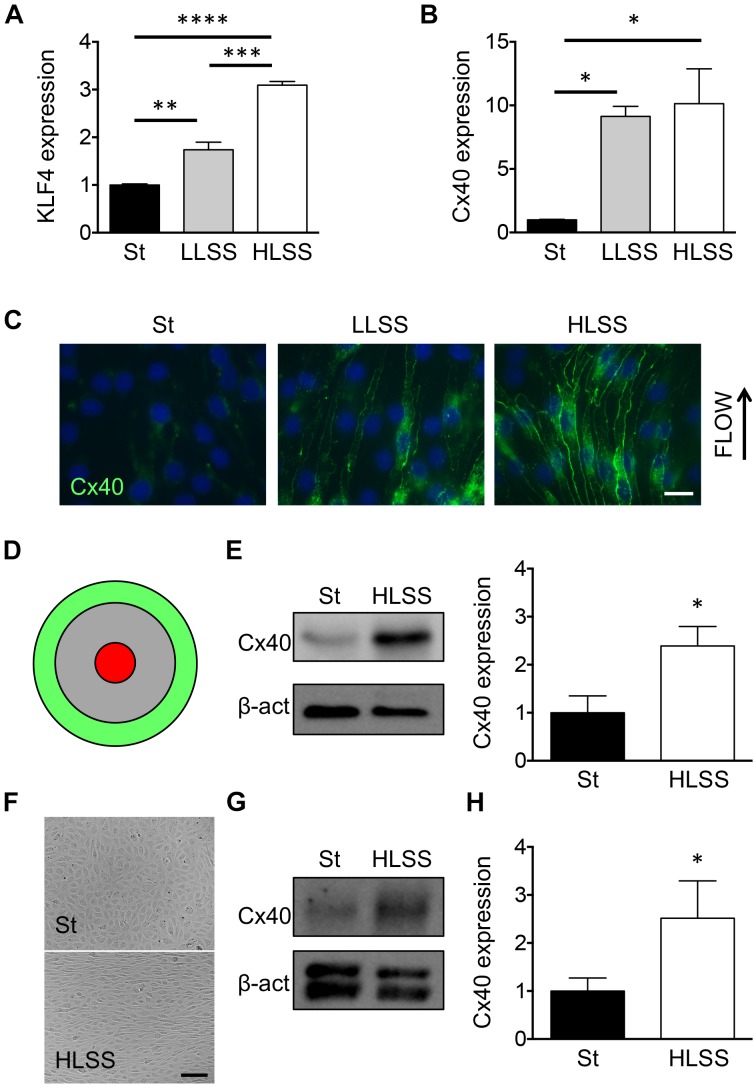
Cx40 expression is gradually regulated by shear stress. **(A)** KLF4 expression in bEnd.3 cells under static conditions (St) or exposed to 24 h of LLSS and HLSS was assessed by qPCR. *N* = 3. **(B)** Cx40 expression in bEnd.3 cells under static conditions (St) or exposed to 24 h of LLSS and HLSS was assessed by qPCR. *N* = 3. **(C)** Representative images of Cx40 expression (green) in bEnd.3 cells under static conditions (St) or exposed to LLSS and HLSS for 24 h. Arrow indicates the direction of flow. Nuclei were stained with DAPI (blue). Scale bar represents 10 μm. **(D)** Schematic representation of flow regions in cell cultures induced by orbital rotation; green = HLSS and red = OSS. **(E)** Cx40 expression in bEnd.3 cells under static conditions (St) or exposed to 48 h of HLSS was assessed by Western blotting. *N* = 5. **(F)** Phase-contrast images of HUVECs under static conditions or exposed for 48 h of HLSS. **(G,H)** Cx40 expression in HUVECs under static conditions (St) or exposed to 48 h of HLSS was assessed by Western blotting **(G)** or qPCR (**H**; *N* = 6).

The KLF2 and KLF4 transcription factors have been shown to act as critical regulators of multiple genes involved in endothelial homeostasis under HLSS (Davies et al., 2013). Although Cx37 expression is regulated by KLF2, this transcription factor seems not involved in the regulation of endothelial Cx40 expression (Pfenniger et al., 2012). To identify the transcription factor involved in flow-dependent Cx40 expression, we used the mouse EC line bEnd.3 known to express all three endothelial connexins (Kwak et al., 2001). Confluent cultures of bEnd.3 cells were exposed to LLSS (5 dynes/cm^2^) or HLSS (20 dynes/cm^2^) for 24 h or kept under static conditions. As expected, KLF4 mRNA levels increased gradually with the level of shear stress with a doubling under LLSS and a threefold increase in response to HLSS (Figure 1A). Compared to static conditions, LLSS and HLSS induced Cx40 transcript by about 10-fold (Figure 1B). In addition, a gradual increase in Cx40 protein was observed in ECs in response to increasing levels of laminar shear stress (Figure 1C). Shear stress-dependent regulation of Cx40 was also investigated using an orbital rotating platform. As detailed elsewhere (Warboys et al., 2014), on a rotating platform ECs at the periphery of the culture well are exposed to HLSS and cells in the center to OSS (Figure 1D). As shown in Figure 1E, HLSS enhanced Cx40 expression compared to static conditions. As expected (Denis et al., 2017), Cx40 expression in bEnd.3 cells was low under OSS and not different from static conditions (data not shown). Finally, we confirmed shear stress-dependent regulation of Cx40 expression in primary human ECs. As expected, HUVECs aligned in the direction of the flow at the periphery of the rotating culture well and were cobblestone-shaped under static conditions (Figure 1F). Moreover, HLSS enhanced Cx40 expression, similar to the effect observed in bEnd.3 cells (Figure 1G,H).

**FIGURE 2:**
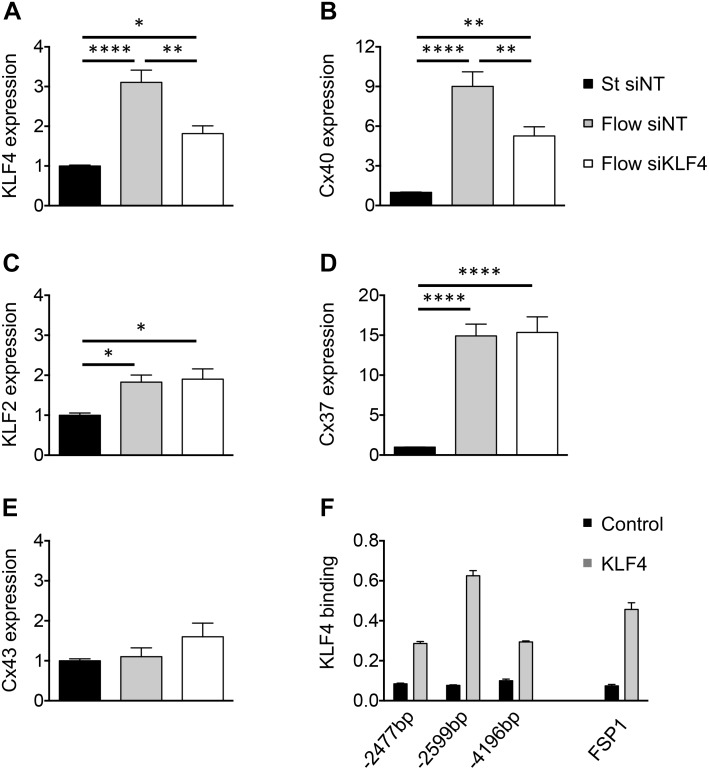
KLF4 regulates flow-dependent Cx40 expression. KLF4 **(A)**, Cx40 **(B)**, KLF2 **(C)**, Cx37 **(D)** and Cx43 **(E)** expression in bEnd.3 cells transfected with KLF4 siRNA or NT siRNA and subsequently exposed to 48 h of HLSS or kept under static conditions. *N* = 6. **(F)** Representative ChIP results for bEnd.3 chromatin precipitated with antibodies against KLF4. Three different CACCC KLF-consensus sites (indicated in bp) were amplified for Cx40 (left) and one for FSP1 (right; positive control). Levels of DNA are normalized to input. *N* = 2.

To determine the possible causal relationship between flow-driven KLF4 and Cx40 expression, confluent cultures of bEnd.3 cells were transfected with siRNA directed against KLF4. Under static conditions, siKLF4 effectively reduced the expression of KLF4 mRNA by 50% compared to siNT (data not shown). Next, confluent ECs were treated with siKLF4 or siNT for 24 h followed by 48 h of HLSS or static conditions. In presence of siNT the ECs responded similarly to HLSS with a threefold induction of KLF4 transcript and a ninefold increase in Cx40 mRNA (compare Figure 2A,B vs. Figure 1A,B). However, a prior exposure to KLF4 siRNA blunted the HLSS-driven induction of both KLF4 and Cx40 by half (Figure 2A,B). Notably, the HLSS-driven induction of KLF2 and Cx37 was not affected by siKLF4 (Figure 2C,D). Moreover, Cx43 transcript was not significantly affected by HLSS or siKLF4 (Figure 2E). Together, these results indicate that KLF4 participates in the induction of Cx40 by HLSS in ECs.

The Cx40 promoter contains multiple CACCC elements, which are preferential binding sites of the KLF transcription factors. To investigate whether the regulation of Cx40 expression by KLF4 is direct or involves other pathways, we performed ChIP on bEnd.3 cells using an antibody directed against mouse KLF4. To ensure the efficacy of KLF4 immunoprecipitation, we first investigated the FSP1 promoter, which is known to be regulated by KLF4 through direct binding (Cuttano et al., 2016). Indeed, we observed an enrichment of the specific FSP1 promoter locus, thus validating this mouse KLF4 antibody for the ChIP procedure (Figure 2F). Similarly, three potential KLF4-binding sites were investigated in the Cx40 promoter region. We observed an enrichment of all three selected promoter loci with the region located 2,599 bp upstream from the transcription start site being mostly enriched compared to control IgG (Figure 2F). Altogether, these results indicate that KLF4 binds directly to at least three loci in the Cx40 promoter.

### Cx40-Dependent Flow-Induced Differential Gene Expression

**FIGURE 3:**
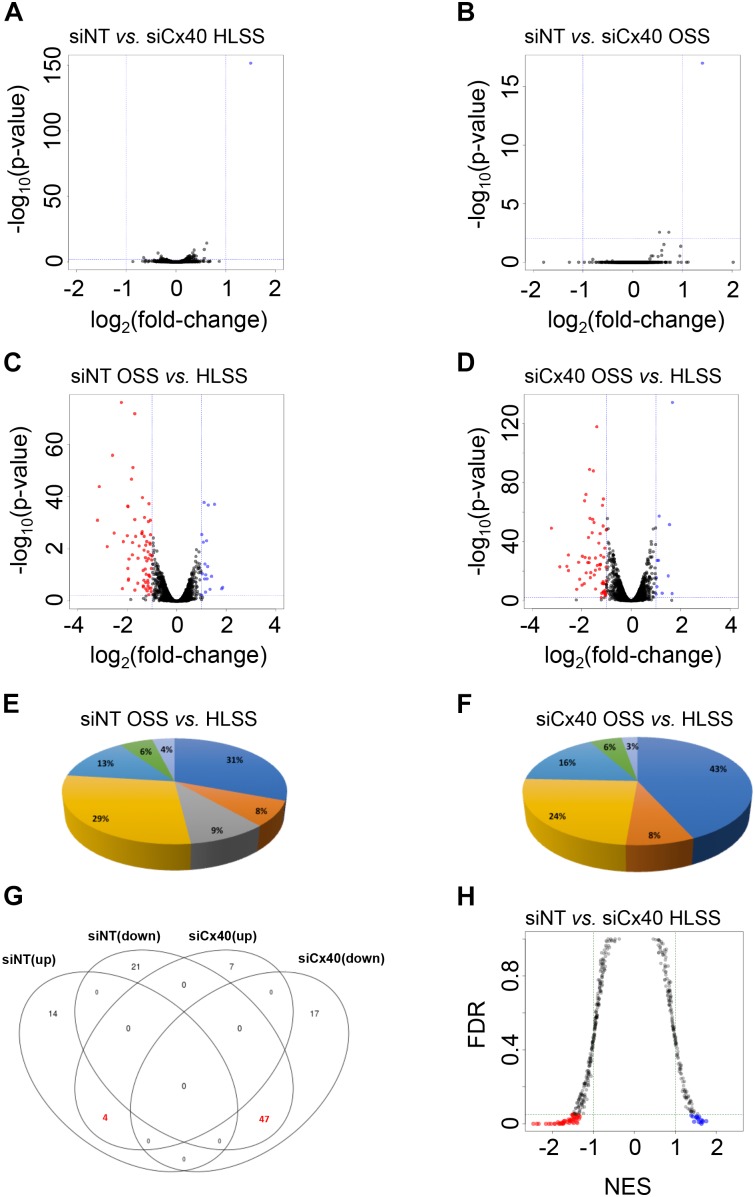
Cx40-dependent flow-induced differential gene expression. **(A–D)** Differential gene expression in bEnd.3 cells exposed to HLSS or OSS after treatment with siNT or Cx40 siRNA. Volcano plots show relationship between degree of gene expression change (log_2_ of fold-change; *x*-axis) and statistical significance of this change (–log_10_ of *P*-value; *y*-axis). Colored dots represent differentially expressed genes (*P*-value < 0.01) with fold-change >2 that are down-regulated (red) or up-regulated (blue). **(A)** Differential gene expression in ECs exposed to HLSS after treatment siCx40 compared to siNT. **(B)** Differential gene expression in ECs exposed to OSS after treatment with siCx40 compared to siNT. **(C)** Differential gene expression in siNT-treated (control) ECs exposed to HLSS compared to OSS. **(D)** Differential gene expression in siCx40-treated ECs exposed to HLSS compared to OSS. **(E,F)** Pie chart representation for differentially expressed genes organized for seven atherosclerosis-related processes, i.e., inflammation (dark blue), ECM interaction (orange), permeability (gray), angiogenesis (yellow), metabolic processes (light blue), vascular tone (green), and immune response (lilac). Differential expressed genes between siNT-treated **(E)** or siCx40-treated **(F)** cells exposed to HLSS compared to OSS. **(G)** Venn diagram representing gene overlap in OSS (as compared to HLSS) with or without Cx40 silencing. Highlighted are 47 and 4 genes that are commonly up- or down-regulated, respectively. **(H)** Gene set enrichment analysis of pathways in bEnd.3 cells exposed to HLSS after treatment with siNT or siCx40. Colored dots represent differentially regulated pathways (FDR < 0.05) with NES > 1 which involve genes that are down-regulated (red) or up-regulated (blue).

Although the importance of Cx40 for the maintenance of a healthy quiescent endothelium has become increasingly clear (Chadjichristos et al., 2010; Denis et al., 2017), no unbiased large-scale functional analysis to detail the participating signaling pathways has been performed until now. To shed light on the Cx40-dependent signaling pathways in the endothelium under various shear stress conditions, we performed RNAseq. First, we compared differential gene expression between HLSS and OSS under control conditions or after knock-down of Cx40. For each comparison between up- and down-regulated genes, genes with a fold-change and *P*-values thresholds higher than 2 and lower than 0.01, respectively, were considered as differentially expressed. Remarkably, comparing bEnd.3 cells exposed to similar shear stresses but silenced or not for Cx40 resulted in a significant differential expression of only one gene out of the approximately 10,450 tested, namely Gja5 (encoding Cx40) (Figure 3A,B). This not only illustrates the effective silencing of Cx40 but also validates the reproducibility of the experiment. As expected, under control conditions (siNT) OSS induced a considerable change in the gene expression profile with 18 genes being up-regulated and 68 genes down-regulated when compared to HLSS (Figure 3C and Supplementary Tables 1, 2). Further analyses of these differentially expressed genes revealed groups involved in various atherosclerosis-related processes such as inflammation (16), angiogenesis (15), metabolic processes (7), endothelial permeability (5), ECM interaction (4), vascular tone (3) and immune response (2), as shown in Figure 3E. After effective knock-down of Cx40, the total number of differentially expressed genes under OSS was rather similar, with 11 genes being up-regulated and 64 genes down-regulated when compared to HLSS (Figure 3D and Supplementary Tables 3, 4). However, the relative contribution of genes involved in inflammation increased from 31 to 43% and genes involved in endothelial permeability were no longer differentially expressed after silencing of Cx40 (Figure 3F). Finally, we observed a considerable overlap between the differentially expressed genes under OSS (as compared to HLSS) with or without Cx40 silencing. As shown in Figure 3G, 47 genes were commonly down-regulated and four genes commonly up-regulated, thus more than half of the OSS-induced differentially expressed genes were regulated independently from the Cx40 expression level.

Next, we performed GSEA to identify essential pathways that are regulated by Cx40 under specific shear stress conditions. As Cx40 expression level in arterial regions exposed to OSS is rather low and Cx40 expression is down-regulated by OSS *in vitro* (Denis et al., 2017), we decided to focus on the HLSS conditions. Of the 483 pathways tested, 16 were up-regulated and 48 down-regulated when comparing ECs with a knock-down of Cx40 with control ECs under HLSS (Figure 3H and Supplementary Tables 5, 6). Whereas the top enriched up-regulated pathways in control ECs under HLSS show large diversity, the top enriched down-regulated pathways however point rather uniformly to signaling pathways involved in DNA repair and cell cycle control, suggesting a potential role for Cx40 in this process.

### Cx40-Dependent Cell Proliferation

**FIGURE 4:**
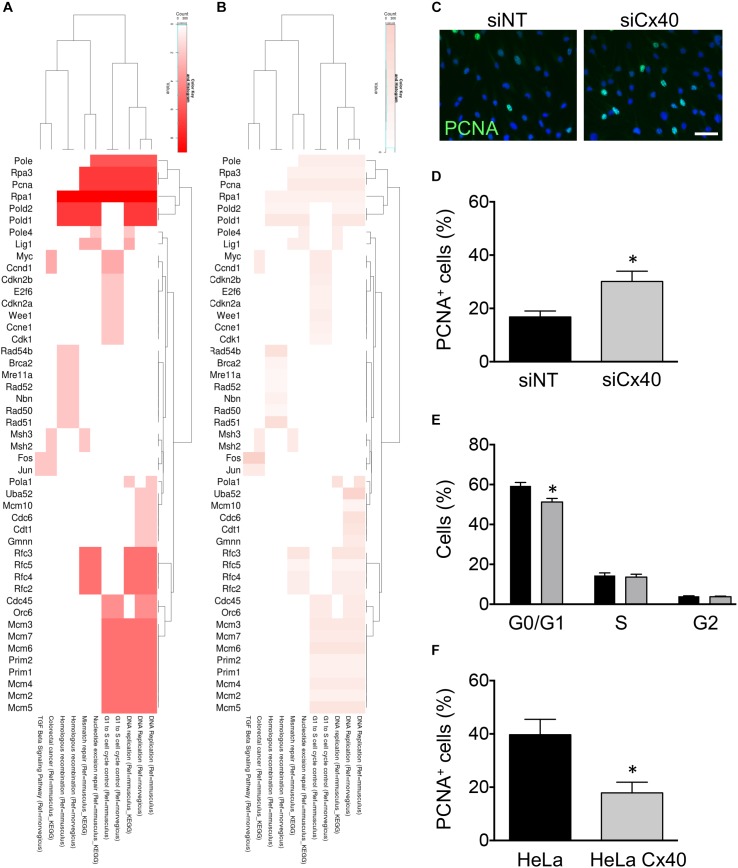
Cx40 controls EC proliferation. **(A,B)** Heat-maps visualizing leading-edge analysis of GSEA results, in which color intensity represents the number of occurrences **(A)** or the fold-change of each gene **(B)**. Adjusted *P*-values were all <0.001. **(C)** Representative images of PCNA expression (green) in bEnd.3 cells after treatment with siNT or siCx40 and exposed to HLSS for 48 h. Nuclei were stained with DAPI (blue). Scale bar represents 50 μm. **(D)** Quantification of B. *N* = 4. **(E)** Cell cycle distribution (G0/G1, S, G2) in bEnd.3 cells exposed to 48 h of HLSS and analyzed by FACS after staining with Hoechst 33342. **(F)** Proportion of PCNA^+^ parental HeLa cells or HeLa cells expressing Cx40. *N* = 4.

The role of Cxs in tumor growth has long been recognized (Aasen et al., 2016). In addition, ECs in straight parts of arteries display a quiescent state (Kwak et al., 2014). To obtain insight in candidate genes that might participate to the regulation of EC proliferation by Cx40, we performed leading-edge analysis of the GSEA results. Extraction of the core members of high scoring gene sets that contributed to the GSEA score identified the Replication proteins a1 and a3 (*Rpa1, Rpa3*), the proliferating cell nuclear antigen (*Pcna*) and DNA polymerases (*Pold1, Pold2*, and *Pole*) as genes with high number of occurrences (Figure 4A) among which *Pcna* displayed a high fold-change (Figure 4B). To investigate whether Cx40 plays a role in the inhibition of proliferation in ECs exposed to HLSS, we treated bEnd.3 cells with siCx40 and siNT for 24 h and subsequently exposed them to 24 h of HLSS. The ECs in which Cx40 was effectively silenced displayed an almost doubled proportion of PCNA-positive cells (Figure 4C,D), indicating an increased proliferation rate. Moreover, FACS-based cell cycle analysis revealed that the proportion of cells in the resting G0 or G1 phase was decreased by 9% in ECs with effective Cx40 silencing (Figure 4E).

To investigate whether the inhibitory effect of Cx40 on EC proliferation could be generalized to other cell types, we used Cx-deficient parental HeLa cells and HeLa cells stably transfected with Cx40. Transfected HeLa cells showed a high expression of Cx40 located at cell–cell contacts and efficient gap junctional coupling following microinjection of fluorescent dye, as reported previously (Denis et al., 2017). Interestingly, the proportion of PCNA-positive cells was reduced to half as compared to parental Cx-deficient HeLa cells (Figure 4F), pointing to a ubiquitous role of Cx40 in cell cycle control. Together, these results suggest that Cx40 regulation by HLSS may be involved in the maintenance of a quiescent endothelium.

### Cx40 Contributes to EC Proliferation *in vivo*

**FIGURE 5:**
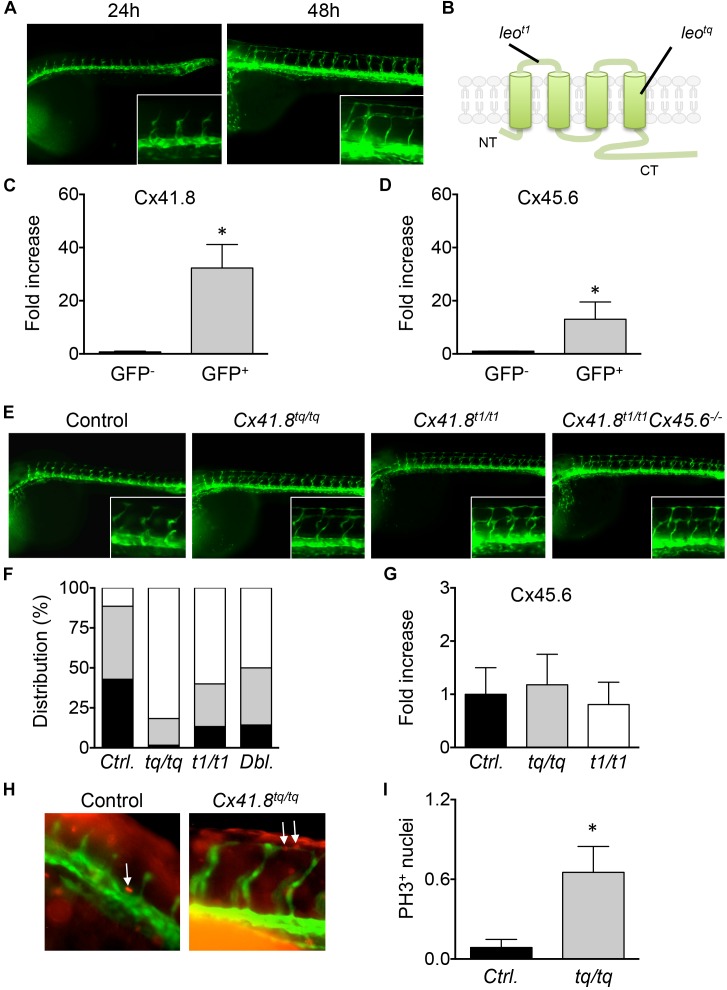
Cx40 contributes to EC proliferation *in vivo*. **(A)** Vascular development in *Tg(flk1:eGFP)* zebrafish embryos at 24 and 48 hpf. Intersegmental vessels (ISVs) sprout from the dorsal aorta (DA) and give rise to the dorsal longitudinal anastomotic vessel (DLAV). **(B)** Schematic representation of location of *leo^t1^* and *leo^tq^* mutation in Cx41.8. **(C,D)** Cx41.8 and Cx45.6 expression in eGFP^+^ and eGFP^-^ cells isolated from *Tg(flk1:eGFP)* zebrafish embryos at 24 hpf was assessed by qPCR. *N* = 3. **(E)** Progress of ISV formation at 28 hpf in *Tg(flk1:eGFP)* (control; *N* = 7), *Tg(flk1:eGFP)Cx41.8^tq/tq^* (*N* = 6), *Tg(flk1:eGFP)Cx41.8^t1/t1^* (*N* = 6) and *Tg(flk1:eGFP)Cx41.8^t1/t1^Cx45.6^-/-^* (*N* = 7) embryos. **(F)** Scores are: partial ISV sprouts (in black), complete ISVs (in gray) and ISVs with complete DLAV branching (in white). **(G)** Cx45.6 expression in eGFP^+^ cells isolated from *Tg(flk1:eGFP), Tg(flk1:eGFP)Cx41.8^tq/tq^* and *Tg(flk1:eGFP)Cx41.8^t1/t1^* embryos at 24 hpf was assessed by qPCR. *N* = 3. **(H)** Examples of PH3 staining (in red) during ISV formation at 24 hpf in *Tg(flk1:eGFP)* and *Tg(flk1:eGFP)Cx41.8^tq/tq^* embryos. Arrows indicate PH3^+^ cells. **(I)** PH3 positive nuclei in proximal ISVs of *Tg(flk1:eGFP)* (control *N* = 23) and *Tg(flk1:eGFP)Cx41.8^tq/tq^* (*N* = 23) embryos at 24 hpf.

During vascular development in zebrafish, small caliber ISVs sprout from the DA by angiogenesis and give rise to the DLAV, processes that require careful orchestration of cell behavior of adjacent ECs (see Figure 5A for examples). The arterial/venous identity of ECs in the developing zebrafish is determined before circulation begins (Lawson and Weinstein, 2002). To investigate the role of endothelial Cx40 on EC proliferation *in vivo*, we used zebrafish with knock-out mutations in *Cx41.8* (t1) and *Cx45.6* genes as well as with a *Cx41.8* (tq) mutation that leads to reduced gap junction channel function (Figure 5B) (Watanabe et al., 2006). These zebrafish Cx40 orthologs both show a high degree of homology to mammalian Cx40 (Supplementary Table 7). We first determined the expression of the Cx40 orthologs in *Tg(flk1:eGFP)* embryos. Thus, cells were dissociated from 24 hpf embryos and FACS-sorted, and the expression of Cx41.8 and Cx45.6 was assessed in eGFP^+^ and eGPF^-^ populations by qPCR. We found that Cx41.8 and Cx45.6 transcripts were, respectively, 32 and 13 times enriched in eGFP^+^ cells, indicating considerable expression of these Cxs in the endothelium of zebrafish embryos (Figure 5C,D). Next, we compared the progress of ISV formation at 28 hpf in *Tg(flk1:eGFP), Tg(flk1:eGFP)Cx41.8^t1/t1^, Tg(flk1:eGFP)Cx41.8^tq/tq^* and *Tg(flk1:eGFP)Cx41.8^t1/t1^Cx45.6^-/-^* embryos. Whereas only 11% of ISVs in *Tg(flk1:eGFP)* control embryos displayed completed branching for DLAV formation, this was enhanced to over 50% in all *Cx41.8*/*Cx45.6* mutant embryos (Figure 5E,F). Interestingly, the *Cx41.8^tq/tq^* mutant showed most advanced vascular development at 28 hpf (Figure 5E,F), suggesting that channel-dependent rather than channel-independent effects are involved in the mechanism linking Cx40 to cell proliferation. Of note, compensatory upregulation of Cx45.6 expression in *Cx41.8^t1/t1^* and *Cx41.8^tq/tq^* mutants was excluded as their mRNA level for this Cx was not different from *Tg(flk1:eGFP)* controls (Figure 5G). To investigate the effects of Cx40 channel-dependent intercellular communication on cell proliferation *in vivo*, we performed immunostainings for the proliferation marker PH3 in *Tg(flk1:eGFP)* and *Tg(flk1:eGFP)Cx41.8^tq/tq^* embryos (Figure 5H). The number of PH3-positive cells was significantly higher in the ISVs of *Tg(flk1:eGFP)Cx41.8^tq/tq^* embryos than in *Tg(flk1:eGFP)* controls, suggesting that the enhanced speed of ISV formation in *Tg(flk1:eGFP)Cx41.8^tq/tq^* embryos is, at least in part, due to increased proliferation of ECs (Figure 5I). Overall, our study demonstrates that Cx40 is a critical regulator of EC proliferation *in vitro* and *in vivo*, a function that appears preserved during evolution.

## Discussion

Using *in vitro* experiments and a large-scale functional analysis, we confirm here that Cx40 expression is regulated by shear stress, being gradually up-regulated by laminar shear stress. We demonstrated that flow-induced Cx40 expression is directly controlled by the transcription factor KLF4. Using RNAseq and GSEA, cell cycle progression was identified as an important downstream target of Cx40 under HLSS. The functional role of Cx40 in the control of cell proliferation was supported by further *in vitro* studies and in a zebrafish model. Overall, our study demonstrates that Cx40 is a critical regulator of EC proliferation *in vitro* and *in vivo*, thereby likely contributing to endothelial quiescence in healthy arteries and restraining vascular development.

KLF2 and KLF4 are key controllers of endothelial homeostasis in large arteries (Atkins and Jain, 2007). Endothelial-specific ablation of both *Klf2* and *Klf4* genes in adult mice demonstrated the absolute requirement of these two KLFs to maintain normal endothelial physiology and vessel integrity in the adult animal (Sangwung et al., 2017). We have previously shown in EC cultures that KLF2 directly binds to the Cx37 promoter and induces its expression under HLSS (Pfenniger et al., 2012). Reduction of KLF2 *in vitro* did not affect Cx40 expression. In this study, we show that KLF4 binds to the Cx40 promoter and gradually regulates its expression in response to increasing levels of shear stress. Of note, KLF4 silencing did not affect Cx37 expression. The KLF-specific regulation of endothelial Cxs *in vitro* is mirrored by the responses of these Cxs to carotid casting *in vivo*. Indeed, Cx37 expression was low in regions of imposed LLSS as well as OSS, however, Cx40 expression was only down-regulated in the OSS region (Pfenniger et al., 2012; Denis et al., 2017). The reason and significance of the different KLF2-Cx37 *vs.* KLF4-Cx40 induction set-points for arterial physiology remains to be investigated. In this respect, it is noteworthy that hypermethylation of the KLF4 promoter has been observed in regions with disturbed blood flow (Jiang et al., 2014). Indeed, flow-dependent epigenomic DNA methylation patterns alter endothelial gene expression, thereby regulating vascular biology and disease (Dunn et al., 2015). Whether, the differential levels of promoter methylation may explain the gradual induction of KLF4 expression by laminar shear stress remains to be investigated as well.

Small metabolites conveyed through gap junctions but also channel-independent effector functions of the cytosolic C-terminal domain (CT) of Cxs control crucial cell processes, including cell growth, in physiology and disease (Naus and Laird, 2010; Kibschull et al., 2015; Leithe et al., 2018). Tissue ischemia activates a cascade of vasodilatory, inflammatory and remodeling processes and endothelial Cxs are increasingly recognized to orchestrate these events. Indeed, Cx37-deficient mice were shown to recover ischemic hind limb function faster and to a greater extent than wild-type animals (Fang et al., 2012). Cx37 appeared to limit recovery from ischemic hind limb injury by exerting growth suppressive effects on arteriogenic and angiogenic responses to ischemia (Fang et al., 2011). In contrast, Cx40-deficient mice exhibited profound and rapid failure of limb survival after an ischemic insult (Fang et al., 2012) due to compromised regulation of tissue perfusion, vascular remodeling and a prolonged inflammatory response (Fang et al., 2013). Targeting endothelial Cx40 in mice reduces angiogenesis in the developing retina and in a tumor setting (Alonso et al., 2016; Haefliger et al., 2017). Although Cx40 expression levels are not affected in Cx37 knock-out mice (Wong et al., 2006), Cx37 expression levels are severely reduced in Cx40-deficient mice (Chadjichristos et al., 2010; Meens et al., 2015), making it impossible to ascribe the above-described effects in Cx40-deficient animals exclusively to this endothelial Cx. Here, we used a RNA silencing rather than a knock-out approach to down-regulate Cx40 expression and this procedure did not affect the expression levels of Cx37, as demonstrated in the RNAseq data (fold-change Gja4: 1.017). Thus, our study permits to investigate the selective function of Cx40 in EC growth. The RNAseq/GSEA revealed significant up-regulation of pathways related to cell cycle control in ECs with knock-down of Cx40 exposed to arterial flow rates (Figure 3H, 4A,B and Supplementary Table 6) and involved genes encoding for replication proteins DNA polymerases and PCNA. Moreover, we showed that the proliferation rate was increased in ECs treated with siCx40 under these flow conditions (Figure 4C,D). Interestingly, proliferation rate of HeLa cells was decreased after transfection of Cx40 in this Cx-deficient cell line (Figure 4F). This illustrates not only that the inverse relation between Cx40 levels and cell proliferation may be extended to another (epithelial) cell type, but also that this relation is independent of which promoter was used for induction of Cx40 expression. In further support of this hypothesis are preliminary experiments using the silent heart zebrafish model *Tg(flk1:eGFP) sih^-/-^* showing at 48 hpf similar Cx41.8 and Cx45.6 expression levels in absence of blood flow than in littermate *Tg(flk1:eGFP) sih^+/-^* and *Tg(flk1:eGFP) sih^+/+^* controls with beating heart (data not shown). Taken together, our data show that Cx40 is a critical regulator of the inhibition of EC proliferation. Whether the regulation of EC proliferation in healthy arteries depends on the synchronization of endothelial responses via gap junctions or channel-independent effects of Cx40 remains to be investigated.

Growth control by Cxs, in particular Cx43, does not always correlate with the extent of gap junctional communication (Moorby and Patel, 2001; Qin et al., 2002). Moreover, ectopic expression of Cx43-CT in, for example, mouse neuroblastoma cells suppressed the growth of these cells to a similar extent as expression of full-length Cx43 (Moorby and Patel, 2001). Mechanistically, it has been shown that Cx43-CT promotes the degradation of S phase kinase-associated protein 2 (Skp2), which subsequently affects p27 degradation and cell growth (Zhang et al., 2003). In addition, direct interactions of Cx43-CT allowing for crosstalk with regulators of cell growth, including β-Catenin, CCN3, c-Src and Cyclin E, have been previously found (Leybaert et al., 2017; Leithe et al., 2018). Interestingly, Cx43 mRNA undergoes alternative translation to generate N-terminal truncated isoforms (Smyth and Shaw, 2013; Salat-Canela et al., 2014). An increasing number of pathophysiological roles are currently ascribed to this Cx43-20k isoform, including actin- and microtubule-based protein transport and epithelial–mesenchymal transition (Basheer et al., 2017; Fu et al., 2017; James et al., 2018). Knowledge on Cx40-CT interacting proteins is limited and whether alternative translation of Cx40 mRNA occurs is presently a matter of debate.

In addition to channel-independent effects, synchronization of cellular responses via gap junctions has also been demonstrated to control cancer cell growth (Chandrasekhar et al., 2013). Mechanistically, intercellular redistribution of cAMP throughout a cell population by open Cx26 gap junction channels appeared to eliminate the cell cycle-dependent oscillations in cAMP in individual cells, thereby inhibiting cell cycle progression. By acting as a pathway to “sink” cAMP, gap junctional communication may thus provide a general means to limit the mitotic rate of cell populations (Chandrasekhar et al., 2013). As cAMP has been shown to permeate Cx40 channels (Kanaporis et al., 2008), a gap junction channel-dependent effect of Cx40 on cell growth inhibition in healthy arterial endothelium may involve a similar mechanism. In line with this hypothesis, zebrafish embryos knock-out for Cx40 orthologs showed faster vascular development, an effect that was further strengthened by potential dominant negative effects of the Cx41.8^tq^ mutant on gap junctional channels formed by other Cxs in the endothelium (Figure 5E,F). Moreover, preliminary experiments on low-density cultures seem not to recapitulate the Cx40-dependent proliferation inhibition observed in HeLa transfectants (data not shown).

Continued research efforts are performed to develop pharmacological strategies aimed at preventing excessive EC proliferation in for instance diabetic retinopathy or tumor growth. Recent studies, including ours, have demonstrated that Cx40 may represent an attractive novel target for the development of molecules that promote endothelial quiescence.

## Author Contributions

J-FD, MD, FM, AH, TL, and SL performed the experiments and/or analyzed the data. BK designed the research. MW, SL, and JB participated to the design of some experiments. J-FD and BK wrote the manuscript. All co-authors corrected the manuscript.

## Conflict of Interest Statement

The authors declare that the research was conducted in the absence of any commercial or financial relationships that could be construed as a potential conflict of interest.

## Footnotes

1 https://www.genomatix.de

2 http://www.wikipathways.org/index.php/WikiPathways

3 http://www.genome.jp/kegg/
