# Supplementary material for: KLF4-Induced Connexin40 Expression Contributes to Arterial Endothelial Quiescence
Source: Front Physiol. 2019 Feb 12;10:80. doi: 10.3389/fphys.2019.00080 (PMC6379456; doi:10.3389/fphys.2019.00080)
Supplement: Supplementary file 1 [file Table_1.DOCX]

## Supplemental tables

Table 1: Down-regulated genes in siNT OSS *vs.* siNT HLSS

| Gene_id | Gene_name | logFC | PValue |
| --- | --- | --- | --- |
| ENSMUSG00000064023 | Klk8 | -3.19 | 1.19E-31 |
| ENSMUSG00000027375 | Mal | -3.12 | 1.51E-44 |
| ENSMUSG00000060063 | Alox5ap | -2.80 | 1.40E-21 |
| ENSMUSG00000043873 | Chil5 | -2.59 | 1.59E-56 |
| ENSMUSG00000037358 | 4930578C19Rik | -2.52 | 9.55E-27 |
| ENSMUSG00000022055 | Nefl | -2.23 | 9.11E-77 |
| ENSMUSG00000056895 | Hist3h2ba | -2.19 | 1.79E-05 |
| ENSMUSG00000026327 | Serpinb11 | -2.15 | 1.79E-23 |
| ENSMUSG00000029121 | Crmp1 | -1.99 | 9.09E-26 |
| ENSMUSG00000020435 | Osbp2 | -1.97 | 4.35E-37 |
| ENSMUSG00000019872 | Smpdl3a | -1.97 | 3.22E-13 |
| ENSMUSG00000027107 | Chrna1 | -1.96 | 7.54E-37 |
| ENSMUSG00000034936 | Arl4d | -1.95 | 1.08E-08 |
| ENSMUSG00000034738 | Nostrin | -1.94 | 3.09E-09 |
| ENSMUSG00000038009 | Dnajc22 | -1.92 | 8.22E-17 |
| ENSMUSG00000026399 | Cd55 | -1.82 | 2.17E-47 |
| ENSMUSG00000041193 | Pla2g5 | -1.79 | 1.98E-18 |
| ENSMUSG00000015709 | Arnt2 | -1.78 | 4.02E-23 |
| ENSMUSG00000029082 | Bst1 | -1.76 | 7.57E-52 |
| ENSMUSG00000029322 | Plac8 | -1.69 | 1.80E-72 |
| ENSMUSG00000030050 | Gkn1 | -1.69 | 6.46E-32 |
| ENSMUSG00000004885 | Crabp2 | -1.69 | 6.19E-05 |
| ENSMUSG00000039616 | Mocos | -1.66 | 1.77E-25 |
| ENSMUSG00000024210 | Ip6k3 | -1.57 | 1.46E-07 |
| ENSMUSG00000026278 | Bok | -1.56 | 3.93E-17 |
| ENSMUSG00000041439 | Mfsd6 | -1.52 | 8.29E-22 |
| ENSMUSG00000027254 | Map1a | -1.40 | 1.58E-26 |
| ENSMUSG00000023411 | Nfatc4 | -1.40 | 1.24E-26 |
| ENSMUSG00000030428 | Ttyh1 | -1.39 | 2.46E-27 |
| ENSMUSG00000053062 | Jam2 | -1.38 | 2.41E-40 |
| ENSMUSG00000042190 | Cmklr1 | -1.35 | 1.96E-12 |
| ENSMUSG00000000244 | Tspan32 | -1.35 | 1.53E-06 |
| ENSMUSG00000041012 | Cmtm8 | -1.35 | 3.50E-06 |
| ENSMUSG00000072964 | Bhlhb9 | -1.32 | 1.08E-08 |
| ENSMUSG00000040543 | Pitpnm3 | -1.32 | 3.21E-20 |
| ENSMUSG00000026875 | Traf1 | -1.31 | 1.70E-25 |
| ENSMUSG00000032246 | Calml4 | -1.30 | 9.68E-33 |
| ENSMUSG00000054203 | Ifi205 | -1.27 | 5.68E-13 |
| ENSMUSG00000006642 | Tcf23 | -1.26 | 2.32E-17 |
| ENSMUSG00000044734 | Serpinb1a | -1.25 | 7.20E-08 |
| ENSMUSG00000021403 | Serpinb9b | -1.25 | 4.15E-22 |
| ENSMUSG00000043613 | Mmp3 | -1.24 | 2.14E-05 |
| ENSMUSG00000038400 | Pmepa1 | -1.23 | 5.78E-11 |
| ENSMUSG00000000416 | Cttnbp2 | -1.21 | 1.64E-23 |
| ENSMUSG00000063415 | Cyp26b1 | -1.20 | 4.20E-16 |
| ENSMUSG00000032068 | Plet1 | -1.19 | 1.27E-04 |
| ENSMUSG00000079853 | Klra1 | -1.17 | 1.19E-08 |
| ENSMUSG00000031557 | Plekha2 | -1.16 | 1.58E-06 |
| ENSMUSG00000047261 | Gap43 | -1.16 | 1.32E-19 |
| ENSMUSG00000048126 | Col6a3 | -1.15 | 9.89E-08 |
| ENSMUSG00000031995 | St14 | -1.14 | 5.54E-17 |
| ENSMUSG00000040249 | Lrp1 | -1.14 | 3.59E-31 |
| ENSMUSG00000035202 | Lars2 | -1.12 | 3.57E-03 |
| ENSMUSG00000037820 | Tgm2 | -1.12 | 5.34E-38 |
| ENSMUSG00000032060 | Cryab | -1.12 | 9.85E-11 |
| ENSMUSG00000011463 | Cpb1 | -1.12 | 1.77E-10 |
| ENSMUSG00000054675 | Tmem119 | -1.11 | 1.20E-05 |
| ENSMUSG00000072620 | Slfn2 | -1.09 | 5.25E-23 |
| ENSMUSG00000017652 | Cd40 | -1.08 | 4.96E-10 |
| ENSMUSG00000053835 | H2-T24 | -1.08 | 1.72E-18 |
| ENSMUSG00000079018 | Ly6c1 | -1.07 | 1.84E-22 |
| ENSMUSG00000075602 | Ly6a | -1.06 | 1.15E-31 |
| ENSMUSG00000001739 | Cldn15 | -1.06 | 3.93E-26 |
| ENSMUSG00000021456 | Fbp2 | -1.04 | 1.11E-05 |
| ENSMUSG00000063851 | Rnf183 | -1.03 | 2.41E-04 |
| ENSMUSG00000032012 | Pvrl1 | -1.02 | 3.22E-13 |
| ENSMUSG00000022847 | Thpo | -1.01 | 1.88E-16 |
| ENSMUSG00000026826 | Nr4a2 | -1.00 | 4.30E-18 |

Table 2: Up-regulated genes siNT OSS *vs.* siNT HLSS

| Gene_id | Gene_name | logFC | PValue |
| --- | --- | --- | --- |
| ENSMUSG00000029641 | Rasl11a | 1.86 | 5.55E-06 |
| ENSMUSG00000030159 | Clec1b | 1.82 | 2.05E-05 |
| ENSMUSG00000030865 | Chp2 | 1.52 | 8.72E-38 |
| ENSMUSG00000021367 | Edn1 | 1.41 | 3.28E-10 |
| ENSMUSG00000023391 | Dlx2 | 1.35 | 3.13E-05 |
| ENSMUSG00000053626 | Tll1 | 1.27 | 1.74E-37 |
| ENSMUSG00000019997 | Ctgf | 1.25 | 6.00E-14 |
| ENSMUSG00000020363 | Gfpt2 | 1.22 | 3.03E-09 |
| ENSMUSG00000037621 | Atoh8 | 1.21 | 8.01E-24 |
| ENSMUSG00000028195 | Cyr61 | 1.17 | 8.65E-11 |
| ENSMUSG00000025784 | Clec3b | 1.16 | 3.75E-04 |
| ENSMUSG00000023092 | Fhl1 | 1.14 | 6.54E-15 |
| ENSMUSG00000040289 | Hey1 | 1.13 | 3.29E-09 |
| ENSMUSG00000032172 | Olfm2 | 1.10 | 1.75E-38 |
| ENSMUSG00000020173 | Cobl | 1.08 | 2.96E-23 |
| ENSMUSG00000030790 | Adm | 1.03 | 3.49E-11 |
| ENSMUSG00000031936 | Hephl1 | 1.01 | 3.42E-26 |
| ENSMUSG00000027533 | Fabp5 | 1.01 | 5.86E-03 |

Table 3: Down-regulated genes siCx40 OSS *vs.* siCx40 HLSS

| Gene_id | Gene_name | logFC | PValue |
| --- | --- | --- | --- |
| ENSMUSG00000027375 | Mal | -3.22 | 8.18E-50 |
| ENSMUSG00000020614 | Fam20a | -2.88 | 9.76E-24 |
| ENSMUSG00000060063 | Alox5ap | -2.59 | 5.06E-24 |
| ENSMUSG00000064023 | Klk8 | -2.53 | 1.18E-31 |
| ENSMUSG00000004885 | Crabp2 | -2.53 | 4.08E-21 |
| ENSMUSG00000019890 | Nts | -2.20 | 1.90E-08 |
| ENSMUSG00000037358 | 4930578C19Rik | -2.12 | 1.02E-26 |
| ENSMUSG00000019872 | Smpdl3a | -2.08 | 2.82E-15 |
| ENSMUSG00000034936 | Arl4d | -1.98 | 2.97E-11 |
| ENSMUSG00000015709 | Arnt2 | -1.97 | 7.24E-31 |
| ENSMUSG00000031375 | Bgn | -1.89 | 2.66E-12 |
| ENSMUSG00000026399 | Cd55 | -1.88 | 1.89E-68 |
| ENSMUSG00000029121 | Crmp1 | -1.86 | 4.54E-30 |
| ENSMUSG00000026875 | Traf1 | -1.82 | 1.01E-72 |
| ENSMUSG00000038400 | Pmepa1 | -1.81 | 1.41E-19 |
| ENSMUSG00000042190 | Cmklr1 | -1.75 | 1.36E-26 |
| ENSMUSG00000054203 | Ifi205 | -1.72 | 1.51E-43 |
| ENSMUSG00000040543 | Pitpnm3 | -1.70 | 1.53E-26 |
| ENSMUSG00000041439 | Mfsd6 | -1.69 | 1.35E-29 |
| ENSMUSG00000043873 | Chil5 | -1.68 | 1.33E-89 |
| ENSMUSG00000022055 | Nefl | -1.66 | 1.75E-56 |
| ENSMUSG00000026327 | Serpinb11 | -1.65 | 2.41E-16 |
| ENSMUSG00000041193 | Pla2g5 | -1.63 | 8.99E-20 |
| ENSMUSG00000027254 | Map1a | -1.57 | 8.61E-47 |
| ENSMUSG00000032060 | Cryab | -1.55 | 7.56E-56 |
| ENSMUSG00000021403 | Serpinb9b | -1.52 | 1.18E-88 |
| ENSMUSG00000029082 | Bst1 | -1.50 | 1.00E-53 |
| ENSMUSG00000020435 | Osbp2 | -1.49 | 3.78E-18 |
| ENSMUSG00000039616 | Mocos | -1.44 | 2.07E-21 |
| ENSMUSG00000063415 | Cyp26b1 | -1.44 | 1.27E-29 |
| ENSMUSG00000024011 | Pi16 | -1.44 | 1.28E-08 |
| ENSMUSG00000029322 | Plac8 | -1.39 | 1.55E-118 |
| ENSMUSG00000027107 | Chrna1 | -1.37 | 1.33E-24 |
| ENSMUSG00000006958 | Chrd | -1.30 | 9.18E-40 |
| ENSMUSG00000031995 | St14 | -1.28 | 3.98E-22 |
| ENSMUSG00000023411 | Nfatc4 | -1.27 | 3.08E-25 |
| ENSMUSG00000030050 | Gkn1 | -1.25 | 3.65E-46 |
| ENSMUSG00000032012 | Pvrl1 | -1.24 | 5.67E-25 |
| ENSMUSG00000006642 | Tcf23 | -1.24 | 2.12E-25 |
| ENSMUSG00000030717 | Nupr1 | -1.23 | 1.89E-31 |
| ENSMUSG00000069270 | Hist1h2ac | -1.21 | 7.85E-03 |
| ENSMUSG00000014543 | Klra17 | -1.19 | 1.01E-05 |
| ENSMUSG00000079853 | Klra1 | -1.17 | 1.54E-12 |
| ENSMUSG00000037820 | Tgm2 | -1.16 | 2.48E-65 |
| ENSMUSG00000024210 | Ip6k3 | -1.14 | 1.66E-06 |
| ENSMUSG00000017652 | Cd40 | -1.13 | 1.36E-13 |
| ENSMUSG00000025856 | Pdgfa | -1.13 | 2.40E-51 |
| ENSMUSG00000026414 | Tnnt2 | -1.13 | 1.46E-12 |
| ENSMUSG00000075602 | Ly6a | -1.12 | 1.15E-69 |
| ENSMUSG00000038009 | Dnajc22 | -1.12 | 1.02E-06 |
| ENSMUSG00000048779 | P2ry6 | -1.12 | 3.23E-12 |
| ENSMUSG00000025934 | Gsta3 | -1.11 | 3.20E-30 |
| ENSMUSG00000044734 | Serpinb1a | -1.09 | 3.39E-12 |
| ENSMUSG00000048126 | Col6a3 | -1.07 | 1.09E-06 |
| ENSMUSG00000000244 | Tspan32 | -1.06 | 4.13E-05 |
| ENSMUSG00000032246 | Calml4 | -1.06 | 2.60E-36 |
| ENSMUSG00000063767 | S100a7a | -1.06 | 1.09E-07 |
| ENSMUSG00000041012 | Cmtm8 | -1.04 | 9.17E-06 |
| ENSMUSG00000067599 | Klra7 | -1.03 | 2.73E-04 |
| ENSMUSG00000032068 | Plet1 | -1.03 | 1.24E-04 |
| ENSMUSG00000054364 | Rhob | -1.01 | 1.86E-23 |
| ENSMUSG00000079018 | Ly6c1 | -1.01 | 1.05E-48 |
| ENSMUSG00000032420 | Nt5e | -1.00 | 7.40E-12 |
| ENSMUSG00000063011 | Msln | -1.00 | 2.46E-14 |

Table 4: Up-regulated genes siCx40 OSS *vs.* siCx40 HLSS

| Gene_id | Gene_name | logFC | PValue |
| --- | --- | --- | --- |
| ENSMUSG00000053626 | Tll1 | 1.66 | 4.88E-135 |
| ENSMUSG00000001076 | C1ql4 | 1.65 | 1.51E-05 |
| ENSMUSG00000030865 | Chp2 | 1.55 | 2.55E-52 |
| ENSMUSG00000038624 | Nepn | 1.50 | 1.54E-17 |
| ENSMUSG00000006235 | Epor | 1.25 | 8.76E-06 |
| ENSMUSG00000031936 | Hephl1 | 1.13 | 4.86E-58 |
| ENSMUSG00000046080 | Clec9a | 1.10 | 4.66E-28 |
| ENSMUSG00000040283 | Btnl9 | 1.05 | 5.40E-28 |
| ENSMUSG00000020363 | Gfpt2 | 1.04 | 2.90E-10 |
| ENSMUSG00000022579 | Gpihbp1 | 1.02 | 2.44E-16 |
| ENSMUSG00000021259 | Cyp46a1 | 1.01 | 3.80E-05 |

Table 5: Up-regulated pathways siNT HLSS *vs.* siCx40 HLSS

| internal_ID | Pathway_name | NES | FDR |
| --- | --- | --- | --- |
| Pathway_1124 | Type I diabetes mellitus (Ref=mmusculus_KEGG) | 1.76 | 1.41E-02 |
| Pathway_1065 | Cell adhesion molecules (CAMs) (Ref=mmusculus_KEGG) | 1.67 | 9.71E-03 |
| Pathway_1100 | Olfactory transduction (Ref=mmusculus_KEGG) | 1.66 | 1.69E-02 |
| Pathway_1027 | Cytokine-cytokine receptor interaction (Ref=mmusculus_KEGG) | 1.64 | 0.00 |
| Pathway_1194 | Systemic lupus erythematosus (Ref=mmusculus_KEGG) | 1.64 | 1.93E-02 |
| Pathway_1147 | Morphine addiction (Ref=mmusculus_KEGG) | 1.62 | 8.05E-03 |
| Pathway_948 | Starch and sucrose metabolism (Ref=mmusculus_KEGG) | 1.59 | 3.68E-02 |
| Pathway_1034 | Neuroactive ligand-receptor interaction (Ref=mmusculus_KEGG) | 1.57 | 9.75E-03 |
| Pathway_1192 | Autoimmune thyroid disease (Ref=mmusculus_KEGG) | 1.56 | 2.93E-02 |
| Pathway_1119 | Renin secretion (Ref=mmusculus_KEGG) | 1.55 | 2.81E-02 |
| Pathway_1196 | Allograft rejection (Ref=mmusculus_KEGG) | 1.53 | 4.13E-02 |
| Pathway_1046 | Peroxisome (Ref=mmusculus_KEGG) | 1.51 | 3.53E-02 |
| Pathway_1041 | Regulation of autophagy (Ref=mmusculus_KEGG) | 1.51 | 3.56E-02 |
| Pathway_1043 | Lysosome (Ref=mmusculus_KEGG) | 1.46 | 1.14E-02 |
| Pathway_719 | Calcium Regulation in the Cardiac Cell (Ref=rnorvegicus) | 1.42 | 3.93E-02 |
| Pathway_1053 | Vascular smooth muscle contraction (Ref=mmusculus_KEGG) | 1.39 | 4.49E-02 |

Table 6: Down-regulated pathways siNT HLSS *vs.* siCx40 HLSS

| internal_ID | Pathway_name | NES | FDR |
| --- | --- | --- | --- |
| Pathway_505 | DNA Replication (Ref=mmusculus) | -2.46 | 0.00 |
| Pathway_775 | DNA Replication (Ref=rnorvegicus) | -2.38 | 0.00 |
| Pathway_1009 | DNA replication (Ref=mmusculus_KEGG) | -2.33 | 0.00 |
| Pathway_1016 | Mismatch repair (Ref=mmusculus_KEGG) | -2.15 | 0.00 |
| Pathway_1004 | RNA transport (Ref=mmusculus_KEGG) | -2.08 | 0.00 |
| Pathway_927 | Pyrimidine metabolism (Ref=mmusculus_KEGG) | -2.03 | 0.00 |
| Pathway_1010 | Spliceosome (Ref=mmusculus_KEGG) | -1.95 | 0.00 |
| Pathway_1015 | Nucleotide excision repair (Ref=mmusculus_KEGG) | -1.94 | 0.00 |
| Pathway_1017 | Homologous recombination (Ref=mmusculus_KEGG) | -1.86 | 8.46E-03 |
| Pathway_509 | G1 to S cell cycle control (Ref=mmusculus) | -1.84 | 0.00 |
| Pathway_502 | Retinol metabolism (Ref=mmusculus) | -1.83 | 1.97E-03 |
| Pathway_1035 | Cell cycle (Ref=mmusculus_KEGG) | -1.80 | 0.00 |
| Pathway_624 | Homologous recombination (Ref=mmusculus) | -1.77 | 6.36E-03 |
| Pathway_740 | G1 to S cell cycle control (Ref=rnorvegicus) | -1.77 | 2.10E-03 |
| Pathway_1002 | Ribosome biogenesis in eukaryotes (Ref=mmusculus_KEGG) | -1.75 | 0.00 |
| Pathway_779 | Homologous recombination (Ref=rnorvegicus) | -1.74 | 6.10E-03 |
| Pathway_535 | Hypertrophy Model (Ref=mmusculus) | -1.73 | 2.05E-03 |
| Pathway_720 | Cell cycle (Ref=rnorvegicus) | -1.73 | 1.94E-03 |
| Pathway_767 | Hypertrophy Model (Ref=rnorvegicus) | -1.72 | 1.24E-02 |
| Pathway_633 | Nucleotide Metabolism (Ref=mmusculus) | -1.70 | 1.60E-02 |
| Pathway_675 | Retinol metabolism (Ref=rnorvegicus) | -1.68 | 1.41E-02 |
| Pathway_1175 | Colorectal cancer (Ref=mmusculus_KEGG) | -1.64 | 8.28E-03 |
| Pathway_513 | ErbB signaling pathway (Ref=mmusculus) | -1.60 | 2.37E-02 |
| Pathway_1003 | Ribosome (Ref=mmusculus_KEGG) | -1.59 | 4.50E-03 |
| Pathway_727 | TGF Beta Signaling Pathway (Ref=rnorvegicus) | -1.58 | 1.71E-02 |
| Pathway_702 | ErbB signaling pathway (Ref=rnorvegicus) | -1.58 | 1.81E-02 |
| Pathway_787 | Adipogenesis (Ref=rnorvegicus) | -1.55 | 1.32E-02 |
| Pathway_713 | Translation Factors (Ref=rnorvegicus) | -1.55 | 2.03E-02 |
| Pathway_506 | TGF Beta Signaling Pathway (Ref=mmusculus) | -1.51 | 2.86E-02 |
| Pathway_736 | Nucleotide Metabolism (Ref=rnorvegicus) | -1.51 | 4.81E-02 |
| Pathway_975 | One carbon pool by folate (Ref=mmusculus_KEGG) | -1.51 | 4.89E-02 |
| Pathway_937 | Tyrosine metabolism (Ref=mmusculus_KEGG) | -1.50 | 4.92E-02 |
| Pathway_574 | Adipogenesis genes (Ref=mmusculus) | -1.50 | 1.04E-02 |
| Pathway_689 | Spinal Cord Injury (Ref=rnorvegicus) | -1.48 | 2.56E-02 |
| Pathway_995 | Biosynthesis of antibiotics (Ref=mmusculus_KEGG) | -1.48 | 4.31E-03 |
| Pathway_1064 | ECM-receptor interaction (Ref=mmusculus_KEGG) | -1.47 | 3.87E-02 |
| Pathway_1019 | Fanconi anemia pathway (Ref=mmusculus_KEGG) | -1.46 | 2.72E-02 |
| Pathway_579 | Translation Factors (Ref=mmusculus) | -1.45 | 4.97E-02 |
| Pathway_1061 | Osteoclast differentiation (Ref=mmusculus_KEGG) | -1.44 | 2.08E-02 |
| Pathway_1141 | Parkinson's disease (Ref=mmusculus_KEGG) | -1.43 | 3.63E-02 |
| Pathway_1123 | Non-alcoholic fatty liver disease (NAFLD) (Ref=mmusculus_KEGG) | -1.40 | 1.03E-02 |
| Pathway_461 | Cytoplasmic Ribosomal Proteins (Ref=mmusculus) | -1.39 | 3.22E-02 |
| Pathway_756 | mRNA processing (Ref=rnorvegicus) | -1.39 | 3.48E-02 |
| Pathway_1166 | HTLV-I infection (Ref=mmusculus_KEGG) | -1.39 | 4.18E-03 |
| Pathway_1085 | TNF signaling pathway (Ref=mmusculus_KEGG) | -1.37 | 4.62E-02 |
| Pathway_1187 | Small cell lung cancer (Ref=mmusculus_KEGG) | -1.35 | 4.61E-02 |
| Pathway_1143 | Huntington's disease (Ref=mmusculus_KEGG) | -1.35 | 2.76E-02 |

Table 7: Homology between Cx40 orthologues (BLAST)

| Comparison | Identities | Positives | Gaps |
| --- | --- | --- | --- |
| human Cx40 / mouse Cx40 | 296/358 (83%) | 259/358 (88%) | 0/358 (0%) |
| human Cx40 / zebrafish Cx41.8 | 210/380 (55%) | 259/380 (68%) | 32/380 (8%) |
| human Cx40 / zebrafish Cx45.6 | 211/405 (52%) | 255/405 (62%) | 52/405 (12%) |
